# Crab barrel syndrome: Looking through the lens of type A and type B personality theory and social comparison process

**DOI:** 10.3389/fpsyg.2022.792137

**Published:** 2022-10-14

**Authors:** Burcu Uzum, Yasemin Ozdemir, Semra Kose, Osman Seray Ozkan, Okan Seneldir

**Affiliations:** ^1^Kocaeli Vocational School, Kocaeli University, Kocaeli, Turkey; ^2^Faculty of Business, Sakarya University, Sakarya, Turkey; ^3^Faculty of Business, Istanbul Aydin University, Istanbul, Turkey; ^4^Manyas Vocational School, Bandırma Onyedi Eylul University, Balıkesir, Turkey; ^5^Ali Rıza Veziroglu Vocational School, Kocaeli University, Kocaeli, Turkey

**Keywords:** crab barrel syndrome, stress, conflict, competition behavior, higher self-esteem

## Abstract

**Introduction:**

There are epistemological studies about the main concept, Crab Syndrome. In this context, the study aims to reveal the antecedents of the Crab Syndrome by evaluating the theoretical background of the Crab Barrel Syndrome within the framework of Social Comparison Theory. The main purpose of this study is to identify the precursors of crab barrel syndrome. In line with this main purpose, the study also aims to reveal the relationship between crab barrel syndrome behaviors and type A versus type B personalities, along with the effect of social comparison behaviors. Type A personality represents the personalities of individuals who are stressed, ambitious, competitive, and quickly take action for their aims. Type B, on the other hand, presents the personality types of individuals who are calm, away from competition, and perform their actions slowly.

**Method:**

It is designed quantitatively, employing scales to quantify type A and type B personalities, social comparison, and crab barrel syndrome. Hypotheses are tested using structural equation modeling.

**Result/discussion:**

It is found that there is a positive relation between the type A personality and the crab barrel syndrome, and a negative one between the syndrome and high self-esteem. The findings suggest that by social comparison, type A and type B personality are precursors of crab barrel syndrome. In the study, the theoretical background of the Crab Syndrome was evaluated within the scope of Social Comparison Theory. In this context, examining the relationship between different organizational behavior theories and crab syndrome is important for other studies.

## Introduction

Crab mentality is learned as part of a community’s culture. In societies with this mentality, successful individuals are seen as a threat to those who want to maintain their position and power (Tagle, 2021). Another formulation suggests that those who try to step forward and disrupt the relationship of integrity are punished in order to prevent the deterioration of the social order and preserve the perception of equality in the society (Baldacchino, 2008). Tall Poppy Syndrome, a similar concept that draws attention to social equality, describes a cultural perception that the people, having “high-status,” deserve better place than they have (Marques et al., 2022). Dasborough and Harvey (2017) explain that cutting the tapering Poppy causes the feelings called “malicious happiness” for those in lower positions. Crab Syndrome, on the other hand, means that individuals, who get promotion, experience the feelings, such as stress, jealousy and anxiety when they encounter their rivals. In other words, crab syndrome is not concerned with whether someone else deserves the place they are in, as in Tall Poppy Syndrome. The concept, also called crab barrel, refers to the attitudes and behaviors of individuals who believe that they should be more successful than others, want others to fail, and cannot tolerate their success (Uzum and Ozdemir, 2020). The opinion of the person appears, due to the comparison. The individual struggles to find out the level of performance and personal development by comparing it with other individuals around her or him. This process is based on social comparison theory (Festinger, 1954). The behavior of an individual cannot be evaluated, independently, from her or his personality although there are various personality types. For example, Type A personality wants to obtain a higher level by being ambitious and competitive or when she or he succeeds. Therefore, the essence of the competition is based on compare and contrast. She or he can be stressful, worried, angry and feel frustration (Soubhari and Kumar, 2014) when he fails to manage this process as she or he plans (Chen, 2010). These feelings and thoughts have effect on the behaviors (Ozdemir and Uzum, 2019), and thus, it triggers that the crab syndrome occurs. Miller’s research on the Crab syndrome (2019) is the most comprehensive research, in terms of phenomenological while Soubhari and Kumar (2014) deal with the crab syndrome from a quantitative perspective, along with the work stress. On the other hand, Pegues (2018) focuses on the crab syndrome in the way of workplace incivility behaviors toward black professionals. As you see, there are very little research discussing the crab syndrome. In addition, it has been observed that the social sharing, known as outcome of Tall Poppy Syndrome, is focused (Dasborough and Harvey, 2017). Sternberg (2019) emphasizes the power of creativity and skill development to cope with the consequences of Tall Poppy Syndrome while Pierce et al. (2017) point out the power of successful self-regulation to deal with the results of this syndrome. Marques et al. (2022) examines Social Dominance Theory, authority, political ideology, and self-esteem as antecedents.

This research aims to fill the gap in the Turkish literature, and to shed light on crab syndrome with social comparison theory. The research explains type A and type B personality traits on the axis of social comparison theory, and it is claimed that these two concepts, that is the individual behaviors, can be the antecedent of the crab syndrome in the organizations. It is predicted that the Crab syndrome can cause undesirable results in the organizations. In this context, determining the causes of the phenomenon will help in the development of measures to eliminate the behavior. In addition, from an organizational point of view, it will prevent individuals from wasting their energy on such behaviors.

## Conceptual framework

### Crab barrel syndrome

Crab barrel syndrome (CBS) is a metaphor based on observation of a fisherman catching crabs and putting them into a basket or barrel: the crabs attempt to escape, but they do so by getting on top of each other, pulling others down, and eventually falling back into the basket themselves (Soubhari and Kumar, 2014). The analogy suggests that none of the crabs can actually get out of the basket (Ozdemir and Uzum, 2019). A person with CBS does not want anyone to advance except for himself.

To put it another way: crab barrel syndrome is the thought “If I cannot do it, neither can you,” mixed with jealousy or hatred and turned into behavior (Caples, 2016). It is the display of negative behaviors by a person dominated by jealousy and feeling anxious or worried in the face of others’ success. Crab barrel syndrome can psychologically hurt both the person exhibiting crab mentality and the person being targeted, and results in social affairs in which at least the two parties interact (Ozdemir and Uzum, 2019) and often leads to conflict.

Pegues (2018) investigated the relationship between crab barrel syndrome and variables such as prejudice, competition, working climate, minority status, organizational discrimination, emotional states, psychological capital, and incivility in organizations. In another study, Soubhari and Kumar (2014) examined the relationship between crab barrel syndrome and job satisfaction. Crab barrel syndrome has also played a role as a barrier to social mobility between cultural and ethnic groups. Examining the concept in its broadest form, Miller (2019) investigated the concepts related to crab barrel syndrome with a phenomenological approach. As can be seen, the number of studies on crab barrel syndrome is quite limited.

### Crab barrel syndrome and social comparison

Identification of individuals who are “climbing up” within a society, organization, or group is accomplished by comparing that individual with others. This is where social comparison (SC) theory comes in. Social comparison also functions like a SWOT (Strengths-Weaknesses-Opportunities-Threats) analysis of the individual. While it provides awareness of strengths, it contributes to the recognition and development of weaknesses (Yu-Ting and Qing-Qi, 2020). Social comparison helps to understand the Crab Barrel Syndrome (Pierce et al., 2017). Social Comparison Theory (Festinger, 1954) is based on two assumptions. Does the individual see himself or herself at a higher or lower position of the person he or she compares? As a result of the social comparison process, the answer to the question emphasizes the concept of “self-esteem.” If the answer is higher, it means he or she has a high self-esteem perception, if the answer is lower, he or she has a low self-esteem perception. The individuals, who think that they are at a higher level than others as a result of social comparison, are in accord with their environment and have the ability to cope with the difficulties they encounter. According to this theory, similar goals and abilities among individuals cause competition (Garcia et al., 2013). The more an individual perceives that they are similar to the person they target, the more they worry as a result of the comparison (Festinger, 1954; Vardaman et al., 2016). According to another view, individuals may also target those they see as different from themselves (Appel et al., 2016). The main point here is that the anxiety incited by others’ behaviors can negatively affect the individual’s well-being (Sarangi, 2014). Individuals who cannot develop their own abilities and skills have difficulty digesting the success of their competitors. On the other hand, the person will try to deal with feelings that make them feel worthless and inadequate (low self-esteem) in case he or she believes that he or she is at a lower level than others as a result of social comparison (Appel et al., 2016). We can assert that this is the point where crab barrel syndrome emerges. If the individual who compares themselves with others finds those others more successful, they may show anxiety and fear for their own performance and exhibit behaviors in order to hinder others. Research on human behavior shows that crab barrel syndrome is caused by low self-esteem (Tagle, 2021). In fact, those with crab mentality exhibit their thoughts in behaviors such as belittlement, humiliation, harsh criticism, grudge-holding, jealousy, and hostility toward others (Lith, 2017). It is pointed out that the compulsion to make social comparisons and the anxiety of being inferior to others is a cognitive experience related to competition (Tse et al., 2018). Individuals who believe themselves to be superior to others through social comparison are adaptable and have the ability to cope with the difficulties they encounter (Jones and Paulhus, 2014).

The competition set off by social comparison manifests itself in individuals’ career aspirations, too − perhaps best expressed with the phrase “if I cannot have it, neither can anybody” (Caples, 2016). Crab has a mindset that does not want others to advance and does not like competition which is where crab barrel syndrome becomes a subject of interest in organizational behavior studies and human resources management. Soubhari and Kumar (2014) indicate the causes of crab barrel syndrome to be feelings such as stress, anxiety, jealousy, and disrespect felt when comparing one’s own performance with that of others. These feelings also reduce the level of trust in one’s relationships with others. Perceiving individuals who are undesirable to compete (which they want to suppress) as a threat depends on determining a reference point. The reference point is identified by comparison. Therefore, we can assume that crab barrel syndrome emerges as a result of social comparison. All these inferences lead us to the following hypothesis:

*H1*: There is a relationship between social comparison and the crab barrel syndrome.

### The relationship of crab barrel syndrome with type A and type B personalities

In the organizational behavior literature, there are concepts and personality theories to explain personality such as six-factor personality, dark triple, Holland’s personality types (Lee and Ashton, 2004; Jones and Paulhus, 2014). The features that characterize the individual, namely his personality, can also cause anxiety, fear and stress. Although there are various personality types in the literature, type A and type B personalities, which are associated with stress (Ghasemian and Kumar, 2017; James and Sidin, 2017; Verma and Mansuri, 2018), are frequently addressed in today’s studies. Stress is a person’s reaction to the negativity s/he experiences in his or her environment, and the ability to cope with stress is related to personality traits (Lazarus, 1996; Ayesha, 2020).

Behavioral science defines personality as the reflection of differences in physical, mental, and spiritual characteristics on the behavior and lifestyle of the individual (Durna, 2005). This reflection includes the patterns of thought and behavior displayed in a stable manner (Burger, 2019). Type A and type B personality traits are opposites of each other. People with mostly type A personality traits tend to be fast: they perform actions such as eating, talking, and walking quickly. They are ambitious: upon achieving a goal, they set an even higher goal. Therefore, they are competitive. Competition makes them aggressive, and the feelings of hostility and anger are inherent in their behavior (Luthans, 1995; Kunnanatt, 2003). These characteristics of the type A personality seem to be negative personality traits in the sense that they fuel emotions such as stress, anxiety, and worry. Otherwise, neither type A personality nor type B personality is deemed positive or negative. People with intense type B personality traits do not tend to compete. Unlike type A, they move more slowly. Speed is unimportant to them. Therefore, they lead a calmer and more balanced life and stay away from conflict (Basarangil and Akyildiz Munusturlar, 2018). It is unlikely that all type A and type B personality traits will be reflected in any one individual’s behavior. In other words, we cannot strictly categorize people into type A and type B personalities. An individual may sometimes reveal type A personality traits, and type B at other times. On the individual level, the line between the two personality types is rather blurry, and one can only be said to tend toward type A or type B (Ozsoy, 2013; Akinci et al., 2015).

Considering the characteristics of these two personality types, the study puts forth the following hypotheses for personality structures with a tendency to crab barrel syndrome:

*H*2: There is a relation between personality traits and crab barrel syndrome.

Crab barrel syndrome hinders agreement on goals and methods in organizations or among individuals, rendering conflict unproductive (Ozdemir and Uzum, 2019) and blocking the success of individuals with potential. Consequently, individuals’ motivation may be eroded.

### Model

**Figure 1:**
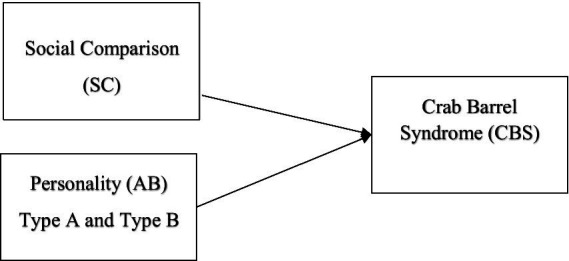
Suggested research model.

The research model is shown in Figure 1. The aim of this study is to discover the antecedents of crab barrel syndrome and understand the degree of relationship between the factors on the left and crab barrel syndrome.

The research model was designed in the light of the hypotheses formed on the basis of the literature review.

## Materials and methods

The purpose of the study is to reveal the relation of crab barrel syndrome with social comparison and type A and type B personalities. To this end, related concepts were presented in the literature review section, hypotheses were formulated, and the research model was chosen.

### Participants and procedure

At the beginning of the research, a questionnaire was tested on a group of 50 people in order to ensure the questions were readable, understandable and conceptually normal. The study proper was then conducted. The convenience sampling technique was chosen in sample selection. Employees actively working in health sectors were included in the research, which examined the attitudes and behaviors of employees with a focus on their personalities and tendencies toward social comparison. It is thought that health sector workers will be exposed to stress more intensely and their self-esteem will be higher, due to the nature of their works.

The purpose of the survey was presented to the employees and they were asked to participate. Ethically necessary explanations and voluntary consent information were presented to the participants, and an online questionnaire was given to those who agreed to participate. The survey was conducted between September 1 and December 30, 2020.

Sample size, significance level (α) and effect size need to be determined in order to calculate a statistical power (Cohen et al., 2003). The sample size to be reached at 80% statistical power, *α* = 0.05 significance level and at 0.30 effect size was calculated as (139 + 139) 278 through the G*Power program in the research. Furthermore, the power calculated as *post hoc* at *α* = 0.05 significance level and at 0.30 effect size was determined as 0.80 (Kyriazos, 2018). Shirsavar et al. (2012) also state that the appropriate sampling interval for CFA or SEM can be calculated *via* the formula (*n*): 5*q* ≤ *n* ≤ 15*q*. On the other hand, it is sufficient for CFA to represent each group with 100 participants in multi-group variables (Kline, 2016). A total of 302 participants were reached. Participants completed all portions of the questionnaires. Therefore, all forms were included in the analyses. SPSS and AMOS programs were employed to evaluate the questionnaires and the model was tested by confirmatory factor analysis (CFA). As an analysis used to evaluate construct validity, CFA tests whether a previously defined construct is correct as a model (Kline, 2016; Orcan, 2018).

### Measures

*Type A and type B personality scale:* The 7-item scale consisting of two opposite poles can be scored 1–8. “I am not competitive / I am competitive.” is an example of an item from the scale. The points obtained are multiplied by three. Those who score below 100 are considered to have a type B personality, and scores above 100 characterize a type A personality. The Turkish adaptation of the scale was made by Ozsoy (2013) (*α* = 0.78).

*Social comparison scale:* Adaptation of the scale into Turkish, the final selection of 18 items was made by Sahin et al. (1993). The scale has a form consisting of two opposite poles and can be scored 1–6. “I feel inadequate / I feel adequate” is an example from the scale. Having a low total score on the scale reveals a low self-esteem, and a high score points to high self-esteem. In this case, the lowest score that can be obtained is 18 while the highest score is 108 (*α* = 0.78). There are opposite statements in both sides of two-side scales. The participant chooses whichever expression he or she thinks is closest to him or her.

*Crab barrel syndrome scale:* The scale was developed by Uzum and Ozdemir (2020). The scale, consisting of five items and a single structure, was designed as a 5-point Likert scale. “That my colleagues are more successful than me makes me anxious” is an example from the scale (*α* = 0.74).

### Findings

A sample size of 200 is suitable for factor analysis (Guilford, 1954). Therefore, the sample of 302 participants in the research is of sufficient size. The demographic structure of the sample is as follows. In terms of gender: 49.7% male (*n* = 150), 50.3% female (*n* = 152). In terms of age: 29.1% (*n* = 88) of the employees are in the 27–33 age range, and 23.8% (*n* = 72) are in the 34–40 age range. In addition, 45.4% (*n* = 137) of the participants had a bachelor’s degree, and 56.6% (*n* = 171) of them are married. Last, 74.8% (*n* = 226) of the participants work in the private sector.

Normality of the data was checked by skewness and kurtosis values. As long as these values are within ±1.5 confidence intervals, the distribution is considered normal (Tabachnick and Fidell, 2019). Correlation, reliability and validity analyzes of the variables in the model have been conducted and the results are presented in Table 1.

**Table 1:** Correlations among variables, mean, standard deviations, and reliabilities.

**Construct**	**1**	**2**	**3**	**Mean**	**SD**	***α***	**AVE CR**
*Social comparison*	1	0.106^*^	−0.129^*^	4.79	0.79	0.93	0.44 0.92
High Self-esteem				5.25	0.51		
Low Self-esteem				4.25	0.42		
*Personality*		1	0.282^*^	4.37	1.49	0.79	0.52 0.84
Personality (A type)				5.16	0.62		
Personality (B type)				3.45	0.49		
*Crab barrel syndrome*			1	2.11	0.76	0.76	0.45 0.75

* *p* < 0.05 (2-tailed); AVE, Average Variance Extracted; CR, Composite Reliable.

It is seen that 162 people have high self-perception while 140 people have low self-perception when the averages of high and low self in the social comparison scale are taken into consideration. Within this scope, it is seen that high self-esteem is higher with an average of 5.25.

It is found that 163 people have type A personality trait while 139 people have type B personality traits when the averages of type A and type B personality in the personality scale are considered. Within this scope, it is detected that type A personality traits are higher with an average of 5.16. It is seen that type B personality (*p* = 0.054) and low self-esteem (*p* = 0.065) have not caused a statistically significant difference in crab syndrome when the analysis results are evaluated.

It has been determined that the goodness of fit values is not in the desired range when the analysis results of the measurement model are examined. The values for analysis are *x*^2^ = 2140.30, *df* = 505; RMSEA = 0.08; CFI = 0.88 and *p* = 0.04; SRMR=0.064. Therefore, SC7 (0.254) and SC13 (0.401) factor loads in the Social Comparison scale have been excluded from the measurement model because they are low. Similarly, it has been found appropriate to exclude the variables AB6 (0.201) and AB7 (0.198) of the personality scale from the measurement. In conclusion, it has been observed that the goodness of fit values is at an acceptable level (Jöreskog and Sörborn, 1993). As a result of the modifications, the proposed fit values were found to be close to the standard fit values (Wolniewicz et al., 2018). The accepted goodness of fit values of the model as found *x*^2^ = 603.84, *df* = 290; RMSEA = 0.06, CFI = 0.91 and *p* = 0.04; SRMR = 0.056). Factor loads are between 0.45 and 0.81.

It is seen that Cronbach’s Alpha coefficients are between 0.76 and 0.93 when the values in the table are taken into account. Since the CR coefficients are between 0.75 and 0.92, internal consistency reliability is ensured. Fornell and Larcker (1981) state that an AVE is less than 0.5 but a CR is greater than 0.6 is sufficient for convergent validity. AVE values between 0.44 and 0.52 in the study reveal convergent validity (Hair et al., 2014).

Suggestions constructed in the model were tested and supported. The H_1_ hypothesis for the effect of social comparison on crab barrel syndrome was supported (*β* = −0.14; *p* = 0.03). High self-scores reduce the tendency of crab barrel syndrome. The H2 hypothesis for type A personality, which was determined by personality score averages, was supported (*β* = 0.12; *p* = 0.01). In addition, the predictive variables explain 22% of the crab syndrome according to the *R*^2^ value.

## Discussion

It has been shown that the type A personality, which is characterized as competitive and ambitious, is more prone to crab barrel syndrome. Pegues (2018) stated that competition and emotional states cause uncivil behaviors and that these variables have a positive relationship with crab barrel syndrome. The findings seem to support Pegues’ (2018) results. Competition-oriented type A personality is found to be positively related to crab barrel syndrome.

The relation between being incline to stress and the crab syndrome as the personality trait of type A has been clarified in line with the comparison theory, and the relation stress and crab syndrome by Soubhari and Kumar (2014) have been extended. Miller (2019) draws attention to the connection of social comparison theory with the crab syndrome. The analysis results have supported the author’s findings in the phenomenological context. Thus, this research differs from other studies in the literature dealing with the crab syndrome.

The small number of studies on crab barrel syndrome narrows the room for comparison. However, the findings of this study indicate that social comparison and type A personality are the precursors of crab barrel syndrome.

Every individual wants to be successful. However, CBS creates barriers to individual and group achievement. This research focused on the individual characteristics of CBS. The relationships between A-type personalities, self-esteem, and CBS were examined. Individuals with type A personality have high stress levels. Stress can cause a person to behave in a negative, discourteous ways, not accepting help and ignoring social norms. Competitive, type A individuals conflict with the person or people who hinder their success because they are ambitious. In this respect, it is seen that type A personality traits are disruptive factor in group settings. Research findings also indicate that anxiety, especially anxiety caused by stress, causes CBS. Pegues (2018) stated that competition and emotional states cause uncivil behaviors and these variables act in the same direction as crab barrel syndrome. The relationship between being open to stress as a type A personality trait and crab barrel syndrome is in line with the findings of Soubhari and Kumar (2014). By focusing on competition instead of cooperation, type A personalities show behaviors that sabotage the success of others with ambition. Miller (2019) determined the effect of social comparison theory on CBS. This finding was supported by the analysis of the current study. High self-esteem does not need an environment of conflict and does not worry about competition. It enables individual performance toward the target with high self-confidence. Conversely, low self-esteem results in low-quality work environments and increases CBS.

## Implications

Highly competitive environments and low level self-esteem trigger CBS. We are thought that the establishment of an effective system of distributive and administrative justice will reduce CBS. It is thought that CBS, which hinders the success of others in environments where competition is intense, can be resolved with merit. Where there is merit, the competition remains within the limits of respectable behavior. It is an important function of human resources managers to express the criteria on which merit will be judged clearly in job interviews. Because CBS includes competition behaviors as well. Human resources managers can determine the attitudes and behavior tendencies of employees by applying personality tests in recruitment. They can also assist in providing social, career, and managerial support. Barriers to career development arising from the structure of the organization can be eliminated with alternatives such as job enrichment, job shaping, rotation, or project-based work. These alternatives strengthen the employee’s feeling of being supported by the management and can increase the self-esteem of the person. In addition, we are thought that the development of different human resources practices such as group activities or leadership games will be effective in reducing the effect of CBS.

## Conclusion

This research demonstrated that social comparison and type A personality as the first sources of CBS motivate practitioners. It indicates that competitive, impetuous, ambitious personalities exhibit low self-esteem and disruptive, conflict-inducing behaviors. It focusses on the individual causes of the behaviors that hinder success and reduce the performance of other individuals in the work environment. The small number of studies on the concept of CBS limits comparisons. However, according to the results of the research, it was determined that social comparison as well as type A and type B personalities were the antecedents of CBS and varied in severity and direction with CBS.

## Limitations and suggestions

Examining all the variables that cause crab barrel syndrome is beyond the scope and capacity of this study. For the sake of applicability, the research focused on a few key variables: social comparison and type A and type B personalities. But there are many other psychological, sociological, and environmental factors that influence crab barrel syndrome. This study was carried out on employees affiliated with a health sector in Turkey however it may be possible to obtain different findings in different sample or social groups. Researching in the larger group and different countries will provide an opportunity to generalize the findings. The research sought to understand the traits of people who exhibited crab barrel syndrome. Conclusions affirm that the two concepts are the precursors of crab barrel syndrome. Future researchers are encouraged to explore the consequences of crab barrel syndrome or its associations with other variables. The proliferation of research on crab barrel syndrome will allow data to be compared.

## Data availability statement

The original contributions presented in the study are included in the article/Supplementary material, further inquiries can be directed to the corresponding author.

## Author contributions

BÜ suggested research model and writing introduction, conceptual framework, implication, conclusion, and discussion. YO suggested Type A and Type B measurement and collected variables. SK analyzed and writing findings. OO general suggestions for manuscript and writing limitations and suggestions. OS writing conclusion. All authors contributed to the article and approved the submitted version.

## Conflict of interest

The authors declare that the research was conducted in the absence of any commercial or financial relationships that could be construed as a potential conflict of interest.

## Publisher’s note

All claims expressed in this article are solely those of the authors and do not necessarily represent those of their affiliated organizations, or those of the publisher, the editors and the reviewers. Any product that may be evaluated in this article, or claim that may be made by its manufacturer, is not guaranteed or endorsed by the publisher.
