# Supplementary material for: Crab barrel syndrome: Looking through the lens of type A and type B personality theory and social comparison process
Source: Front Psychol. 2022 Oct 14;13:792137. doi: 10.3389/fpsyg.2022.792137 (PMC9615545; doi:10.3389/fpsyg.2022.792137)
Supplement: Supplementary file 1 [file Data_Sheet_1.pdf]

Appendix

Please rate the following statements on how you evaluate yourself when you compare yourself with others.

Social Comparison	1	2	3	4	5	6	
1. I feel inadequate.							1. I feel adequate.
2. I feel unskilful.							2. I feel skillful.
3. I feel unlovable.							3. I feel loved.
4. I feel introverted.							4. I feel extrovert.
5. I feel alone.							5. I feel that I am not alone.
6. I feel eliminated							6. I feel accepted.
7. I feel impatient.							7. I feel patient.
8. I feel intolerant.							8. I feel tolerant.
9. I feel obedient.							9. I feel independent.
10. I feel coward.							10. I feel brave.
11. I feel unconfident.							11. I feel confident.
12.I feel shy.							12.I feel outgoing.
13.I feel messy.							13.I feel tidy.
14.I feel inactive.							14.I feel active.
15.I feel hesitant.							15.I feel determined.
16.I feel antipathetic.							16.I feel sympathetic.
17.I feel submissive.							17.I feel contestant.

Type A/Type B Personality	1	2	3	4	5	6	7	8	
1. I pay attention to appointments (dressing, timing etc.).									1. I do not pay attention to appointments (dressing, timing etc.)
2. I am not competitive..									2. I am competitive.
3. Time does not cause pressure on me even if I have an important job.									3. Time causes pressure on me even if I have an important job
4. I can perform only an action at a time.									4. I can perform many actions at a time.
5. I move slowy in most situations (eating, walking etc.).									5. I hurry up in most situations (eating, walking etc.).
6. I do not hesitate to express my feelings (I do not hide my feelings).									6. I hesitate to express my feelings (I hide my feelings).
7. Apart from my job, there are so many things I occupy myself									7. Apart from my job, there are so few things I occupy myself

Crab Barrel Syndrome

1. I want to be the only successful in the organization I work for.
2. That my colleagues are more successful than me scares me.
3. I am a jealous person.
4. I want to my colleagues to be successful than me.
5. That my colleagues are more successful than me makes me anxious.
